# 2479 Acute care research competencies for clinical research professionals: A practitioner inquiry approach and assessment

**DOI:** 10.1017/cts.2018.194

**Published:** 2018-11-21

**Authors:** Jacqueline Knapke, Brett Kissela, Lynn Babcock, Schuckman Stephanie

**Affiliations:** University of Cincinnati

## Abstract

OBJECTIVES/SPECIFIC AIMS: Acute care research is a unique area of clinical research that demands specialized skills, knowledge, and talents from empathetic professionals working in the field. Building off existing competencies for clinical research professionals, the Cincinnati Acute Care Research Council (ACRC) developed additional areas of competency for professionals working in the acute care research discipline. METHODS/STUDY POPULATION: Qualitative data obtained from job shadowing, clinical observations, and interviews were analyzed to understand the educational needs and desires of the acute care research workforce. We then utilized Bloom’s Taxonomy to build acute care research competencies that are measurable for job performance and build off of foundational clinical research professionals’ domains and competencies developed by the Joint Task Force of Clinical Trial Competency. RESULTS/ANTICIPATED RESULTS: Results suggest 35 special interest competencies for acute care clinical research professionals under 8 common domains set by the Joint Task Force of Clinical Trial Competency. Additionally an approved ACRC tactic, from actionable learnings through community assessments throughout 2017, is the creation of a Task Force made up of acute care research Principal Investigators and Clinical Research Directors to focus on the identified training and professional development obstacles in the clinical research enterprise. DISCUSSION/SIGNIFICANCE OF IMPACT: The competencies developed for acute care research should serve as guidelines for training a workforce prepared for the challenges of conducting research with each acute audience, as its own vulnerable population. These competencies will guide development of a multi-pronged program of professional development that will include new hire onboarding, new hire on-job training, and ongoing on-job training.

